# Porphyrins produced by acneic *Cutibacterium acnes* strains activate the inflammasome by inducing K^+^ leakage

**DOI:** 10.1016/j.isci.2021.102575

**Published:** 2021-05-21

**Authors:** Karl-Jan Spittaels, Katleen van Uytfanghe, Christos C. Zouboulis, Christophe Stove, Aurélie Crabbé, Tom Coenye

**Affiliations:** 1 Laboratory of Pharmaceutical Microbiology, Faculty of Pharmaceutical Sciences, Ghent University, 9000 Ghent, Belgium; 2 Laboratory of Toxicology, Faculty of Pharmaceutical Sciences, Ghent University, 9000 Ghent, Belgium; 3 Departments of Dermatology, Venereology, Allergology and Immunology, Dessau Medical Center, Brandenburg Medical School Theodor Fontane, 06847 Dessau, Germany

**Keywords:** Immunology, Microbiology

## Abstract

Some *Cutibacterium acnes* subgroups dominate on healthy skin, whereas others are frequently acne associated. Here we provide mechanistic insights into this difference, using an anaerobic keratinocyte-sebocyte-*C. acnes* co-culture model. An acneic *C. acnes* strain as well as its porphyrins activates NRLP3 inflammasome assembly, whereas this was not observed with a non-acneic strain. Low levels of intracellular K^+^ in keratinocytes stimulated with extracted porphyrins or infected with the acneic strain were observed, identifying porphyrin-induced K^+^ leakage as trigger for inflammasome activation. Using a panel of *C. acnes* strains, we found that porphyrin production and IL-1β release are correlated and are higher in acneic strains. This demonstrates that the latter produce more porphyrins, which interact with the keratinocyte cell membrane, leading to K^+^ leakage, NLRP3 inflammasome activation, and IL-1β release and provides an explanation for the observation that some *C. acnes* strains are associated with healthy skin, whereas others dominate in acneic skin.

## Highlights

• We developed an anaerobic keratinocyte-sebocyte-*C. acnes* co-culture model
• Acneic *C. acnes* strains and their porphyrins activate NRLP3 inflammasome assembly
• Activation of the NRLP3 inflammasome is not observed in non-acneic strains
• Porphyrins from acneic *C. acnes* activate the inflammasome by inducing K^+^ leakage

## Graphical abstract

**Figure:**
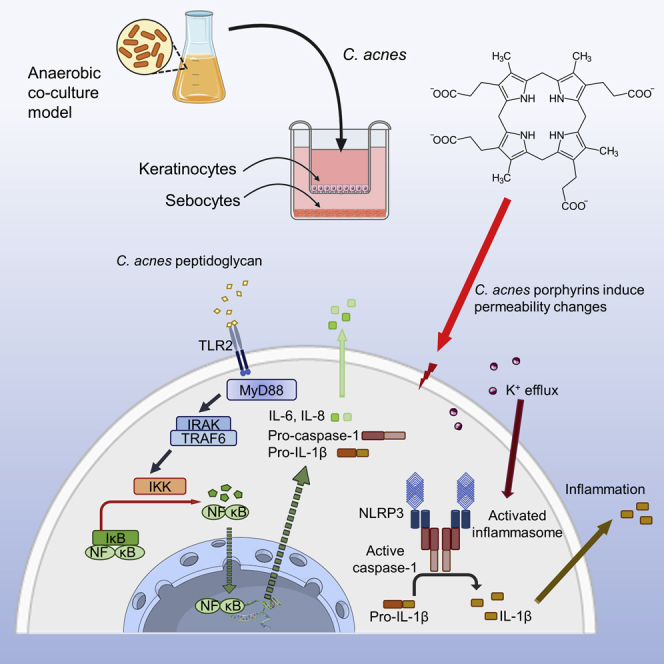

## Introduction

The skin microbiota is an integral part of the skin's first line of defense against colonization and invasion by pathogens and also modulates the immune system (Grice and Segre, 2011). Bacteria such as *Staphylococcus epidermidis* and *Cutibacterium acnes* (formerly known as *Propionibacterium acnes*) (Byrd et al., 2018; Scholz and Kilian, 2016) are commonly part of the healthy skin microbiota. *C. acnes* is a Gram-positive, facultative anaerobic bacterium that can be present in high numbers (up to 10^6^ colony-forming unit/cm^2^) in lipid-rich areas such as the scalp and face (Leyden et al., 1998). However, *C. acnes* colonization inside the skin's pilosebaceous units is thought to play an important role in the pathogenesis of acne vulgaris, a skin disorder affecting 85% of adolescents (Beylot et al., 2014; Heng and Chew, 2020). As *C. acnes* dominates the skin microbiota of both patients with acne and individuals with healthy skin, the exact role of *C. acnes* in the etiology of acne vulgaris still remains unknown (Kwon and Suh, 2016; Shaheen and Gonzalez, 2013; Zouboulis, 2009). The species *C. acnes* consists of different lineages, and this subdivision to some extent separates strains from healthy and acneic skin. Indeed, strains found on acneic skin most often belong to phylotype IA_1_ (which includes ribotypes [RT] 4, 5, and 8), whereas strains not associated with acne or strains associated with healthy skin belong to other phylotypes such as IA_2_, IB, or II (which includes RT 1, 2, 3, 6, and 16). However, the reason behind this link between phylotypes/ribotypes and association with acne is not well understood, and the distinction between *C. acnes* strains recovered from acne samples and those recovered from healthy skin is not absolute—e.g., 20/49 acne samples contained *P. acnes* belonging to RTs 4, 5, and 10, but likewise 9/52 healthy samples did (Fitz-Gibbon et al., 2013).

*C. acnes* can induce the release of proinflammatory cytokines, including interleukin (IL)-1β in human keratinocytes and sebocytes, through activation of the NLRP3 inflammasome (Nagy et al., 2005, 2006; Graham et al., 2004; Li et al., 2014; Huang et al., 2015). This proinflammatory potential of *C. acnes* is thought to be due to the production of host-degrading enzymes, such as lipases, proteases, and hyaluronidases, and other virulence factors, such as the co-hemolytic Christie-Atkins-Munch-Peterson (CAMP) factor and porphyrins (Holland et al., 2010; Nazipi et al., 2017; Lheure et al., 2016; Nakatsuji et al., 2011). The production of porphyrins by *C. acnes* is well known (Xu et al., 2018; Lee et al., 1978; Kjeldstad et al., 1984; Romiti et al., 2000); high levels of porphyrin production by *C. acnes* have been linked to acne severity (Johnson et al., 2016; Barnard et al., 2020; Richter et al., 2016), whereas a decrease in porphyrin levels has been observed in treated acne patients showing clinical improvement (Borelli et al., 2006). In addition, porphyrins could play a role in interspecies interactions and modulate the behavior of other skin bacteria, like *Staphylococcus aureus* (Wollenberg et al., 2014).

In the present study, we investigated whether porphyrin production could be linked to the association of certain *C. acnes* subgroups with acneic versus healthy skin and elucidated how production of porphyrins by acneic *C. acnes* is mechanistically linked to inflammation.

## Results and discussion

### Porphyrins from acne-associated *C. acnes* strain HL053PA1 induce IL-1β release in keratinocytes

**Figure 1:**
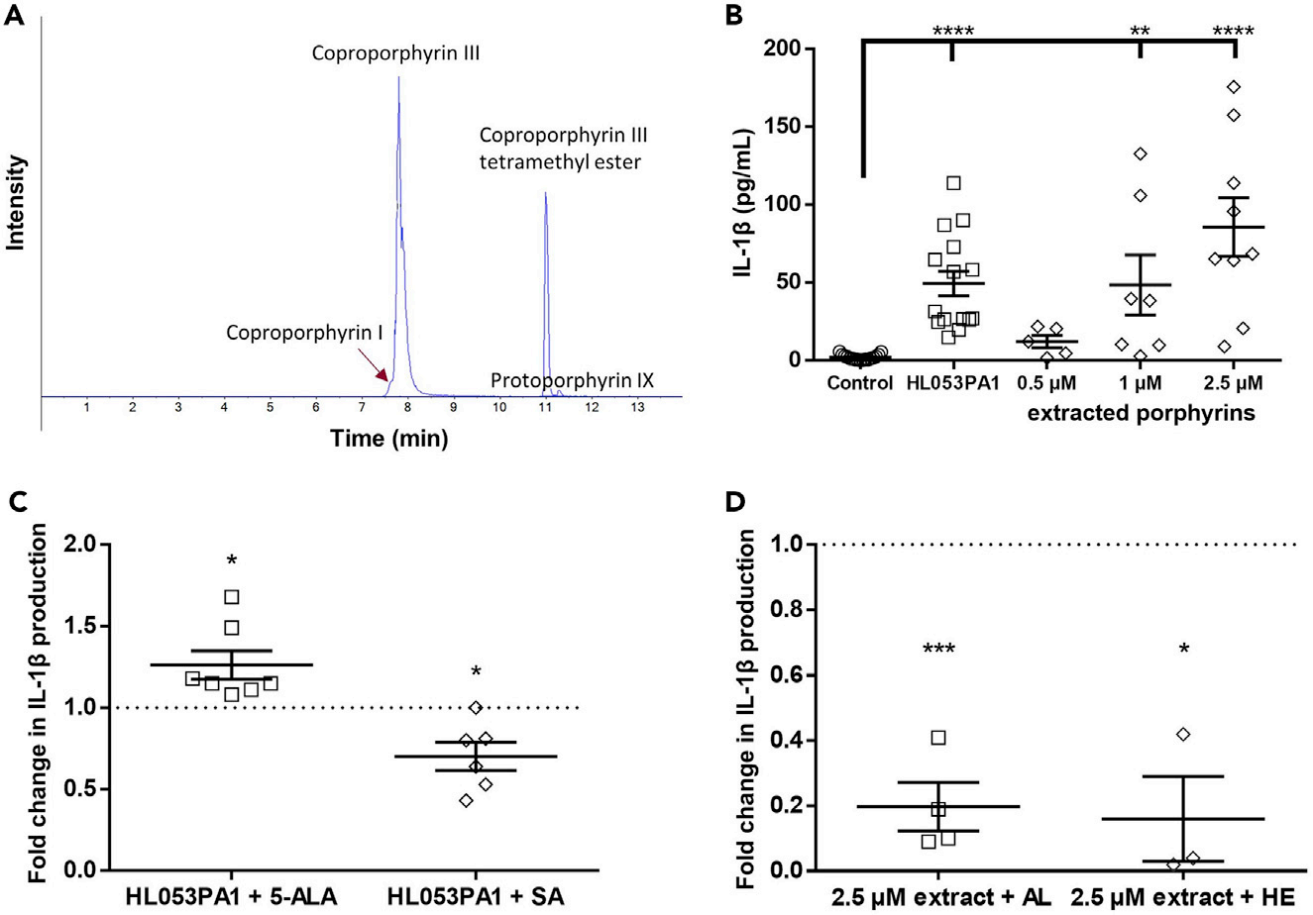
Porphyrin production by *C. acnes* and IL-1β released by HaCaT keratinocytes (A) HPLC analysis of the *C. acnes* HL053PA1 porphyrin extract showing the presence of coproporphyrin I, coproporphyrin III, coproporphyrin III tetramethyl ester, and protoporphyrin IX. (B) Amount of IL-1β released by HaCaT keratinocytes grown in a co-culture model with SZ95 sebocytes after infection with the acneic HL053PA1 strain for 48 h or after priming with 0.1 mg/mL LPS for 4 h followed by exposure to 0.5, 1.0, and 2.5 μM of the extracted porphyrins for 48 h. (C) Fold change in IL-1β release by HaCaT cells, after adding 5-aminolevulinic acid (5-ALA) or succinyl acetone (SA) during infection with the acneic HL053PA1 strain. (D) Fold change in IL-1β release after exposure to extracted porphyrins combined with albumin (AL) or hemopexin (HE), compared with the porphyrins extract only. Error bars represent standard error of the mean. ∗p < 0.05, ∗∗p < 0.01, ∗∗∗p < 0.005, ∗∗∗∗p < 0.001

In order to investigate the inflammatory potential of porphyrins produced by different *C. acnes* strains (Table 1), porphyrins were extracted starting from a bacterial pellet of the acne-associated *C. acnes* strain HL053PA1. High-performance liquid chromatograohy (HPLC) analysis followed by high-resolution mass spectrometry (HPLC-HRMS) revealed several peaks, with coproporphyrin III (CPIII; m/z 655.2767) as the most abundant compound, accompanied by a small amount of coproporphyrin I, presenting as a shoulder on the CPIII chromatographic peak. Based on the area under the curve and assuming similar ionization efficiencies for the different coproporphyrins, CPIII accounted for approximately 75% of the total coproporphyrins. In addition, the presence of coproporphyrin III tetramethyl ester (m/z 711.3391; ∼24%) and protoporphyrin IX (PPIX; m/z 563.2664; ∼1%) could also be demonstrated (Figure 1). These results are in accordance with those of previous studies (Johnson et al., 2016; Borelli et al., 2006; Ashkenazi et al., 2003; Schaller et al., 2005).

**Table 1:** *C. acnes* strains used in this study

Strains	Clade (Fitz-Gibbon et al., 2013)	Ribotype (Fitz-Gibbon et al., 2013)	Phylotype (Mcdowell et al., 2012)	Single locus sequence type (Scholz et al., 2014)	Disease association (Fitz-Gibbon et al., 2013)
**HL072PA1**	IA-1	5	IA_1_	A6	Acne
**HL036PA1**	IA-1	532	IA_1_	A2	Acne
**HL005PA1**	IA-2	4	IA_1_	C2	Acne
**HL038PA1**	IA-2	4	IA_1_	C1	Acne
**HL045PA1**	IA-2	4	IA_1_	C2	Acne
**HL053PA1**	IA-2	4	IA_1_	C2	Acne
**HL056PA1**	IA-2	4	IA_1_	C2	Acne
**HL043PA2**	IA-2	5	IA_1_	C1	Acne
**HL086PA1**	IB-1	8	IA_1_	E4	Acne
**HL002PA1**	IB-2	3	IA_2_	F1	Non-acne
**HL027PA1**	IB-2	3	IA_2_	F1	Non-acne
**HL030PA2**	IB-2	3	IA_2_	F4	Non-acne
**HL059PA1**	IB-2	16	IA_2_	F1	Non-acne
**HL059PA2**	IB-2	16	IA_2_	F1	Non-acne
**HL050PA2**	II	1	II	K4	Non-acne
**HL060PA1**	II	1	II	K1	Non-acne
**HL082PA2**	II	2	II	K6	Non-acne
**HL110PA3**	II	6	II	K2	Non-acne
**HL110PA4**	II	6	II	K2	Non-acne

HaCaT keratinocytes, anaerobically grown in co-culture with SZ95 sebocytes, were infected with *C. acnes* HL053PA1 or exposed to the extracted porphyrins after priming with 0.1 mg/mL lipopolysaccharide (LPS) for 4 h. Compared with the uninfected control, infection with the acne-associated strain HL053PA1 significantly induced the release of IL-1β (p < 0.0001), as did exposure to 1 and 2.5 μM of the extracted porphyrins (p = 0.0077 and p < 0.0001, respectively) (Figure 1). To confirm the actual role of porphyrins in this IL-1β release, HaCaT cells were infected with HL053PA1 alone, or with HL053PA1 in combination with either 5-aminolevulinic acid (5-ALA, 0.2 mg/mL) or succinyl acetone (SA, 0.5 mM), a substrate or inhibitor of the porphyrin biosynthetic pathway, respectively (Johnson et al., 2016; Ebert et al., 1979; Laftah et al., 2008). Addition of 5-ALA significantly increased IL-1β release (p = 0.0229), whereas SA significantly decreased IL-1β release (p = 0.0172), confirming that porphyrins produced by *C. acnes* HL053PA1 play a role in IL-1β release (Figure 1).

The inflammatory potential of these porphyrins was further confirmed by exposing HaCaT cells to porphyrins that had been co-incubated with either albumin or hemopexin (30 min, 1:1 molar ratio). Albumin and hemopexin are porphyrin-binding serum proteins that both contain one specific porphyrin-binding pocket, thus interacting with an apparent stoichiometry of 1:1 (Morgan et al., 1980; Muller-Eberhard and Morgand̊, 1975). A significant decrease in IL-1β release was observed after exposure to porphyrins co-incubated with albumin (p = 0.0017) and hemopexin (p = 0.0232) (Figure 1), again confirming that porphyrins are important in mediating IL-1β release.

### *C. acnes* porphyrins activate the NLRP3 inflammasome

**Figure 2:**
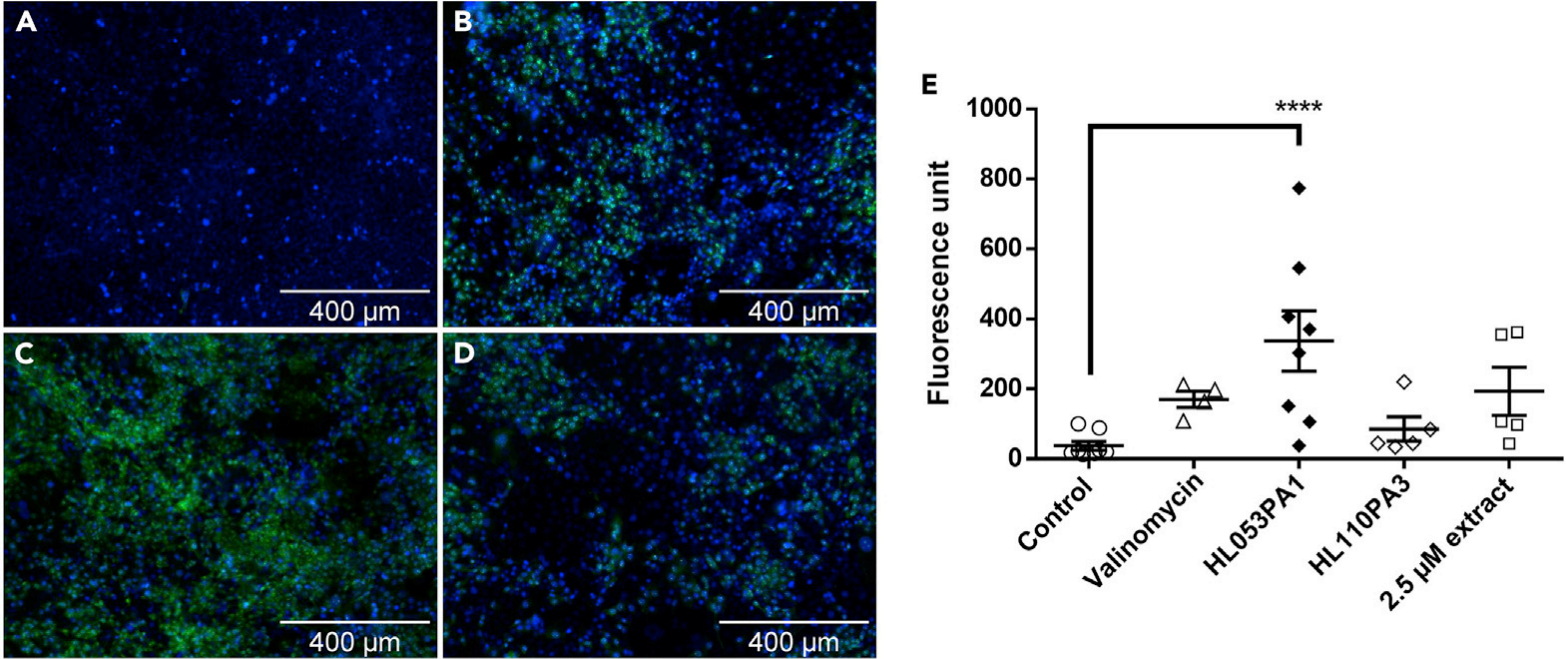
Representative fluorescence microscopy images of HaCaT keratinocytes showing cell nuclei (blue) and active caspase-1 (green) (A–D) (A) Uninfected cells, (B) cells exposed to valinomycin (positive control), (C) cells infected with HL053PA1, and (D) cells exposed to 2.5 μM of the extracted porphyrins for 48 h. Total magnification 300×; scale bars, 400 μm. (E) Quantification of active caspase-1 fluorescence. Error bars represent standard error of the mean. ∗∗∗∗p < 0.001

To further elucidate the mechanism triggering IL-1β release, HaCaT cell monolayers were used. These cells were cultivated in supplemented DMEM for 5 days, after which the medium was changed to supplemented Sebomed for an additional 2 days; supplemented Sebomed contains a high calcium concentration, which enables increased HaCaT cell differentiation, induces sebocyte proliferation, and reduces sebocyte differentiation (Zouboulis et al., 2017; Ottaviani et al., 2020; Micallef et al., 2009). To verify the activation of the NLRP3 inflammasome by *C. acnes* and its porphyrins, caspase-1 activity was measured using a FAM FLICA caspase-1 assay followed by fluorescence microscopy and flow cytometry. Caspase-1 activity was almost undetectable in uninfected HaCaT cells (Figure 2A), whereas valinomycin (positive control, Figure 2B), HL053PA1 (Figure 2C), and porphyrins extracted from HL053PA1 (Figure 2D) induced active caspase-1 after 48 h. Quantification of this fluorescence revealed that the FAM FLICA fluorescence was significantly higher in HaCaT cells infected for 48 h with HL053PA1 (average fluorescence = 336.0, p = 0.0007) compared with the uninfected cells (average fluorescence = 38.1) (Figure 2). Stimulation with valinomycin (average fluorescence = 170.5, p = 0.09) or the extracted porphyrins (average fluorescence = 193.4, p = 0.16) resulted in a non-significant increase in fluorescence.

**Figure 3:**
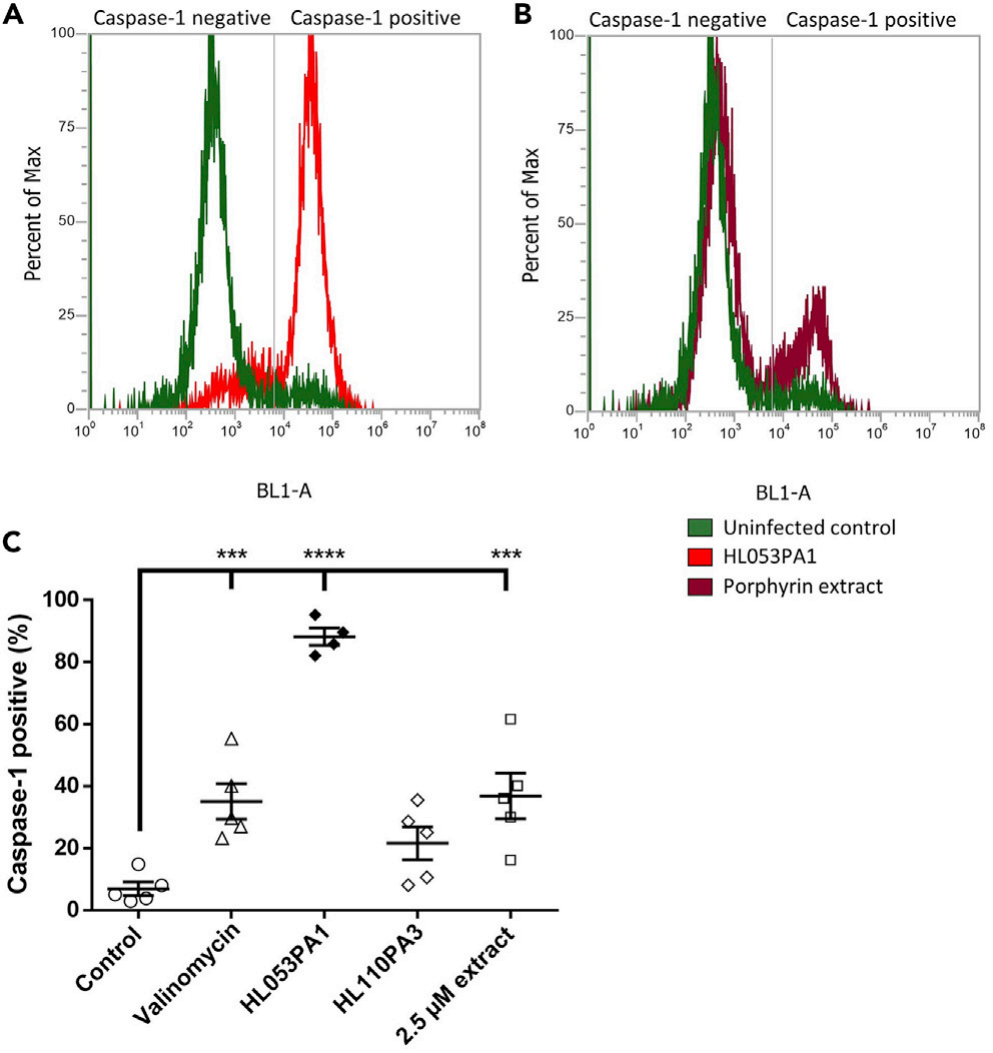
Flow cytometric analysis of caspase-1-positive keratinocytes (A) Population distribution of caspase-1-containing keratinocytes after 48 h of infection with HL053PA1 (red) compared with uninfected cells (green). (B) Population distribution of caspase-1-containing keratinocytes after 48-h exposure to 2.5 μM extracted porphyrins (maroon). (C) Caspase-1-positive population expressed as percentage of total cells in the sample. Error bars represent standard error of the mean. ∗∗∗p < 0.005, ∗∗∗∗p < 0.0001

Next, to quantify the fraction of cells containing active caspase-1, FAM FLICA-stained HaCaT cells were analyzed using flow cytometry. As expected, only 7% of uninfected control cells were caspase-1 positive, whereas 88% of HaCaT cells infected with acne-associated strain HL053PA1 were caspase-1 positive (p < 0.0001); cells exposed to valinomycin and extracted porphyrins also contained a significantly increased population of active caspase-1-containing cells (p = 0.0037 and p = 0.0022, respectively) (Figure 3). These results suggest that porphyrins produced by *C. acnes* act as pathogen-associated molecular patterns that induce IL-1β in keratinocytes through the activation of the NLRP3 inflammasome and the subsequent activation of caspase-1. Indeed, recent studies have shown the activation of the NLRP3 inflammasome and the production of IL-1β by monocytic cells after infection with *C. acnes* (Kistowska et al., 2014; Qin et al., 2014; Sahdo et al., 2013; Guo et al., 2017).

### *C. acnes* porphyrins cause permeability changes and K^+^ efflux in HaCaT keratinocytes

**Figure 4:**
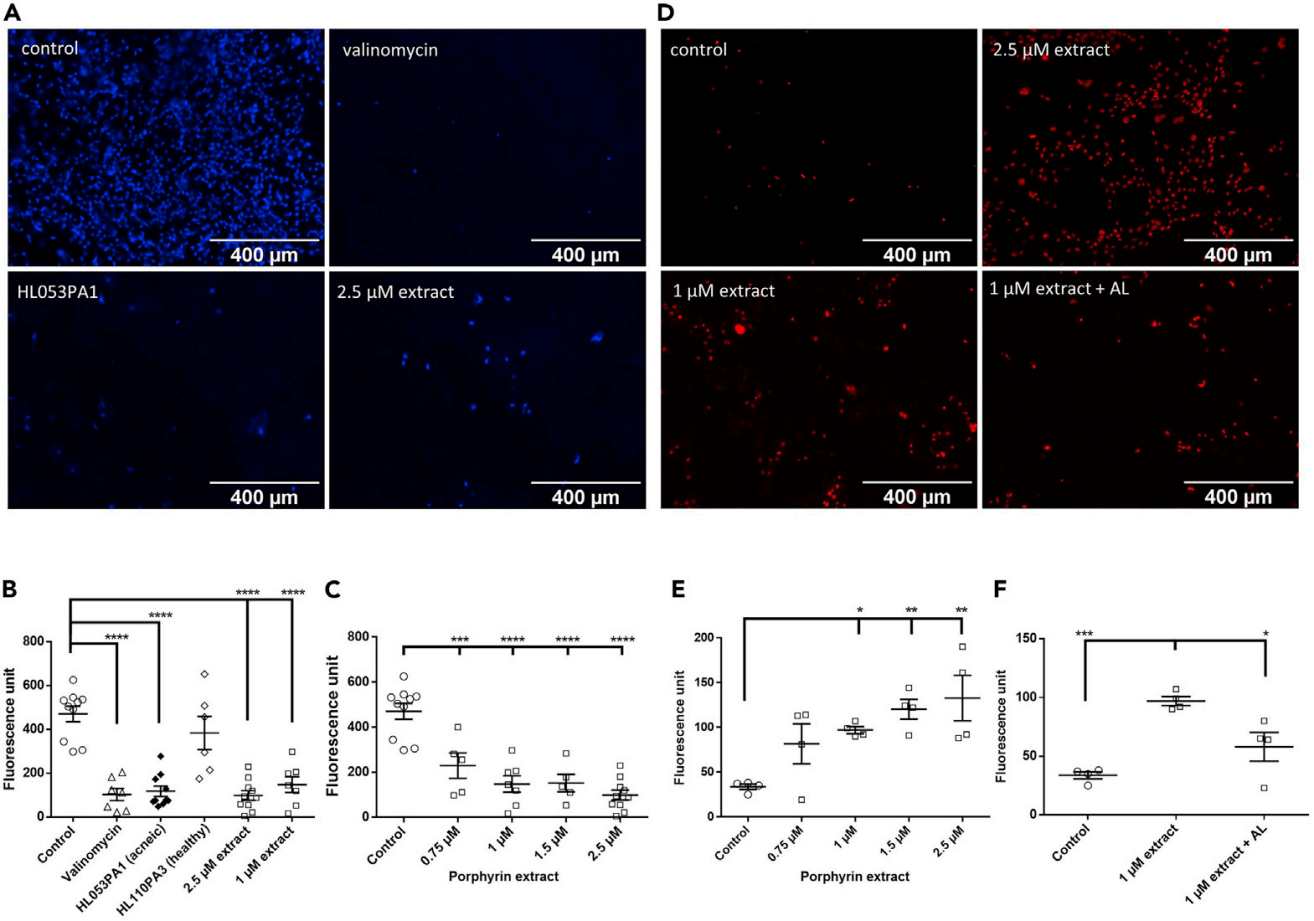
K^+^ efflux and permeability changes of keratinocytes (A) Representative fluorescence microscopy images of HaCaT cells stained with the potassium-sensitive fluorophore PBFI-AM, uninfected, after 48 h of infection with the acneic HL053PA1 strain, or after stimulation with valinomycin or 2.5 μM porphyrins. Scale bars, 400 μm. (B) Quantification of PBFI-AM fluorescence. (C) PBFI-AM fluorescence in HaCaT cells exposed to increasing concentrations of the extracted porphyrins. (D) Fluorescence microscopic images of HaCaT cells stained with propidium iodide (PI) after 30-min exposure to the extracted porphyrin or a combination with albumin. (E) Quantification of PI fluorescence after 30-min exposure to increasing concentrations of porphyrins. (F) PI fluorescence after 30-min stimulation with 1 μM porphyrins or 1 μM porphyrins combined with 1 μM albumin. Error bars represent standard error of the mean. ∗p < 0.05, ∗∗p < 0.01, ∗∗∗p < 0.005, ∗∗∗∗p < 0.001. See also Figures S1 and S2.

**Figure 5:**
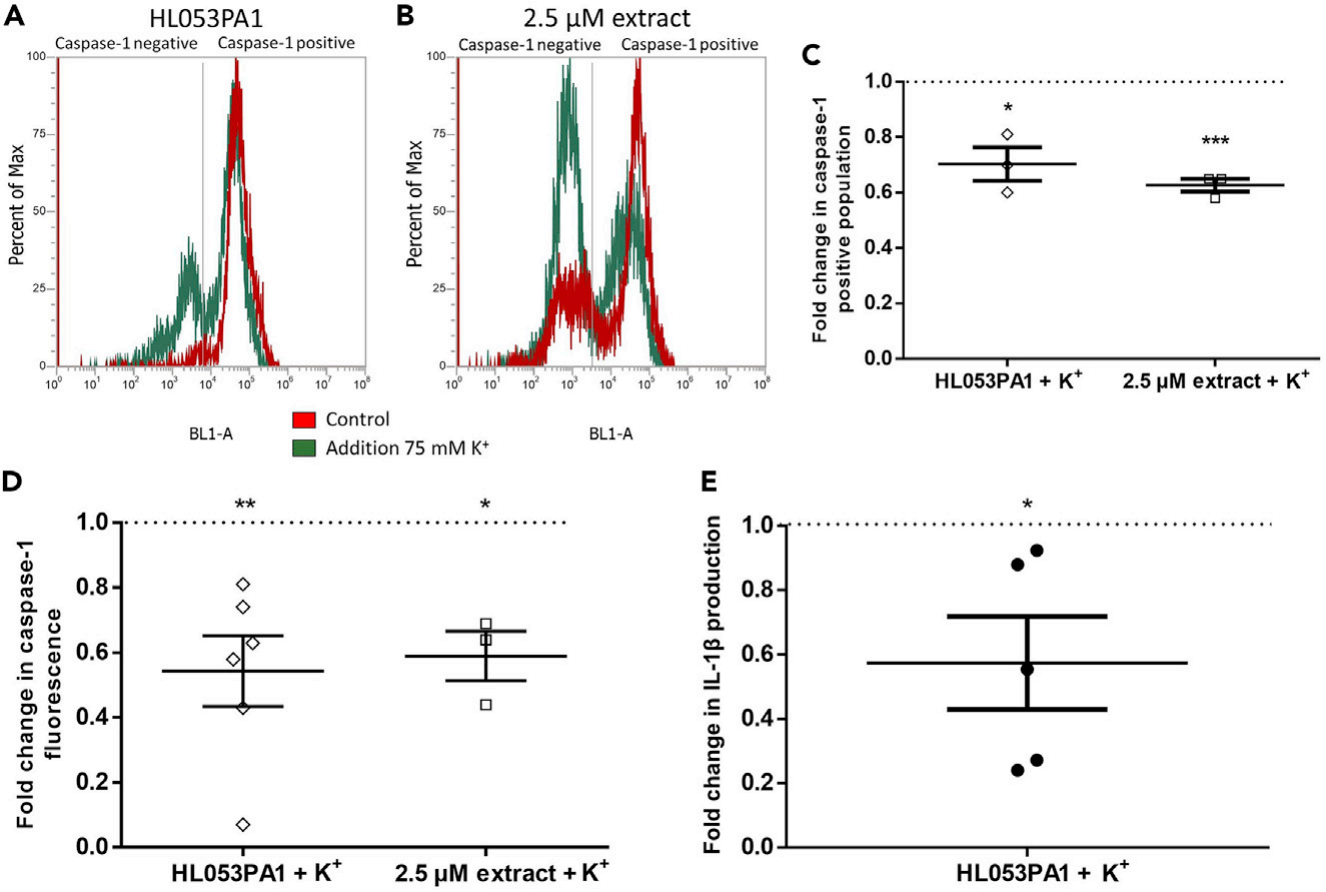
Inhibitory effect of 75 mM extracellular K^+^ on K^+^ efflux and inflammasome activation (A–C) Flow cytometric analysis of active caspase-1 containing HaCaT cells after 48 h of infection with HL053PA1 (A) or exposure to 2.5 μM extracted porphyrins (B) either without (red) or in combination with 75 mM K^+^ (green). (C) Flow cytometric data expressed as caspase-1-positive population in percentage of total cells in the sample. (D) Fold change of fluorescence of active caspase-1 (FAM-YVAD-FMK stained cells) after 48 h of infection with HL053PA1 or exposure to 2.5 μM extracted porphyrins in combination with 75 mM K^+^. (E) Relative IL-1β release in high potassium conditions compared with control. Error bars represent standard error of the mean. ∗p < 0.05, ∗∗p < 0.01, ∗∗∗p < 0.005

K^+^ efflux was determined in HaCaT cells using the fluorescent probe PBFI-AM. Fluorescence microscopy showed high fluorescence in uninfected cells, whereas HaCaT cells treated with the K^+^ ionophore valinomycin or 2.5 μM of the extracted porphyrins or infected with HL053PA1 for 48 h showed little fluorescence, indicating a low intracellular K^+^ concentration (Figure 4). Next, PBFI-AM fluorescence was quantified to confirm the observed K^+^ efflux. Indeed, 48-h infection with HL053PA1 resulted in a significant decrease in fluorescence (p < 0.0001) and exposing the cells to valinomycin or 1 or 2.5 μM of the extracted porphyrins significantly decreased intracellular K^+^ as well (all p < 0.0001) (Figure 4). Additionally, cells exposed to increasing concentrations of the porphyrin extract showed decreasing fluorescence levels in a concentration-dependent manner (Figure 4). This effect was also observed using commercially available CPI, CPIII, and PPIX (concentration: 1 μM) (Figure S1). We subsequently investigated the effect of porphyrins on cell permeability using propidium iodide (PI). Fluorescence microscopy revealed more fluorescent cells after 30 min exposure to the extracted porphyrins compared with the control; as expected this increase was less pronounced after exposure to a mixture of porphyrins and albumin (Figure 4D). Quantification of PI fluorescence of cells exposed to increasing concentrations of the porphyrin extract showed increasing levels of fluorescence in a concentration-dependent manner, in line with results obtained with the PBFI-AM stain. Significantly higher fluorescence was detected after exposing the cells to 1 μM (p = 0.044), 1.5 μM (p = 0.0059), or 2.5 μM (p = 0.0020) of porphyrins (Figure 4). Furthermore, fluorescence decreased again when porphyrins were combined with albumin (Figure 4). To assess cell viability after 30 min exposure to porphyrins, lactate dehydrogenase release was measured. The fraction of dead cells after porphyrin exposure did not differ from that in the control, indicating that the increase in PI fluorescence is not due to killing of cells (Figure S2). To support the hypothesis of K^+^ efflux as the trigger for NLRP3 activation, several experiments were carried out with cells maintained in an environment with an extracellular K^+^ concentration of 75 mM, as previous studies have shown that a high extracellular K^+^ concentration can reduce K^+^ leakage and partly inhibit the assembly of the NLRP3 inflammasome (Thi Tran and Kitami, 2019; Muñoz-Planillo et al., 2013). As shown in Figure 5, a decrease in the HaCaT population containing active caspase-1 was obtained, when cells were either infected with HL053PA1 or exposed to porphyrins in combination with 75 mM K^+^. These results were confirmed by measuring the fluorescence levels of the stained cells (Figure 5). Finally, HaCaT cells grown in the SZ95 co-culture model were infected with HL053PA1 with or without 75 mM K^+^ after which IL-1β release was measured. IL-1β production by the keratinocytes was reduced to approximately 60%, confirming the importance of K^+^ efflux in the assembly and activation of the inflammasome after infection with *C. acnes* (Figure 5).

### The non-acne strain HL110PA3 does not cause caspase-1 activation and K^+^ efflux in keratinocytes

HaCaT cells were infected with the non-acne *C. acnes* strain HL110PA3 but after 48 h no induction of the activation of caspase-1 was observed (Figure 2). In line with this result, flow cytometry analysis revealed only a slight and non-significant increase in active caspase-1-containing cells (p = 0.173) (Figure 3) and no significant decrease in fluorescence was observed using the PBFI-AM probe after 48-h infection with HL110PA3 (p = 0.487) (Figure 4), which means that this strain does not cause K^+^ leakage and therefore cannot trigger the activation of the inflammasome.

### Porphyrin production differs between acne-associated and other *C. acnes* strains and correlates with differential IL-1β but not IL-8 and IL-6 production

**Figure 6:**
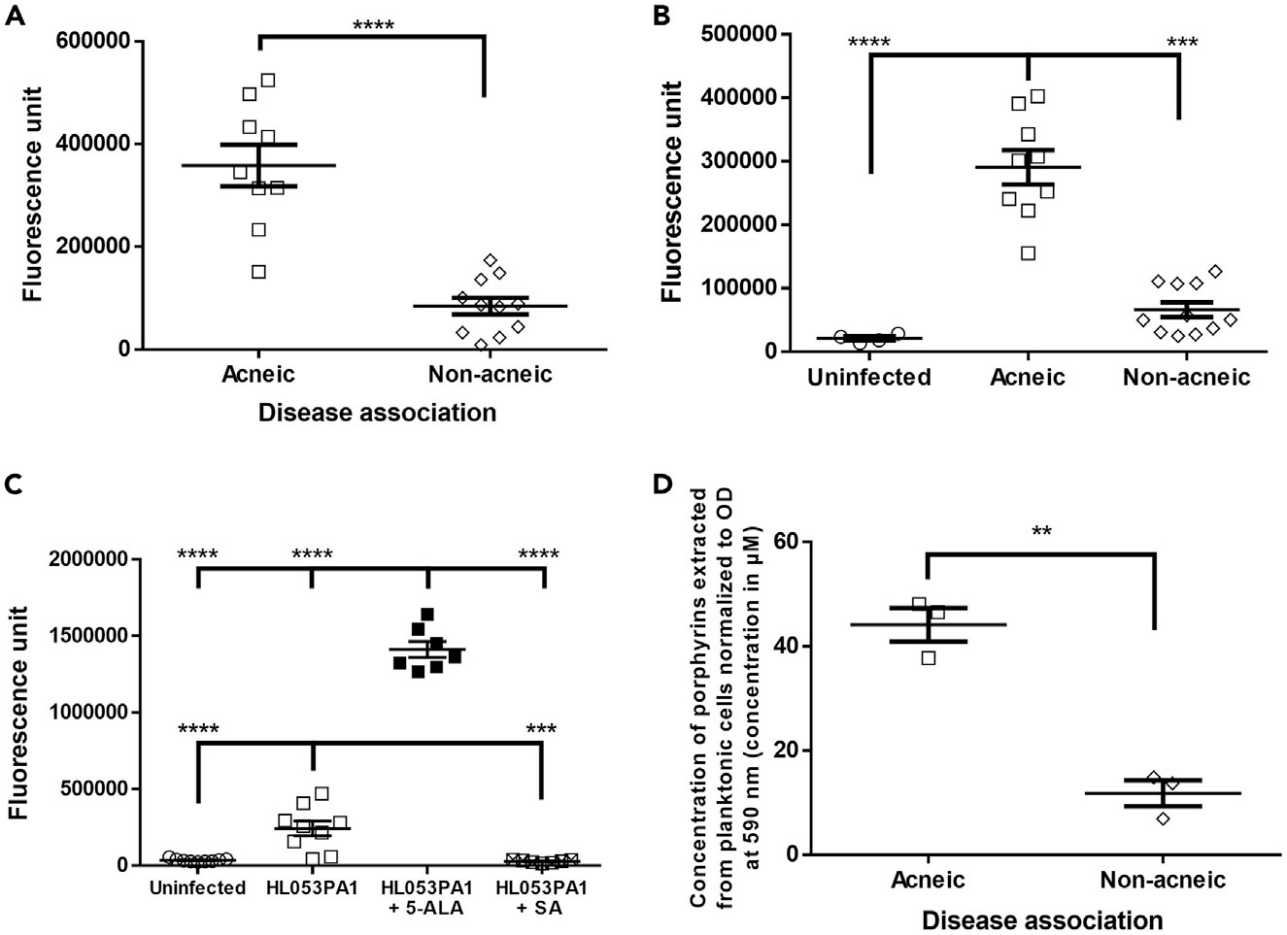
Differential porphyrin production by *C. acnes* strains (A) Porphyrin-derived fluorescence of acne- and health-associated *C. acnes* strains in supernatant of planktonic cultures. (B) Porphyrin-derived fluorescence in supernatant of co-cultures after 48 h of infection with *C. acnes*. (C) Effect of porphyrin pathway substrate (5-ALA) and inhibitor (SA) on porphyrin-derived fluorescence in supernatant during 48 h of infection with acneic strain HL053PA1. (D) Quantification of total porphyrins extracted from acne- and health-associated *C. acnes* strains using HPLC-HRMS. Error bars represent standard error of the mean. ∗∗p < 0.01, ∗∗∗p < 0.005, ∗∗∗∗p < 0.001

**Figure 7:**
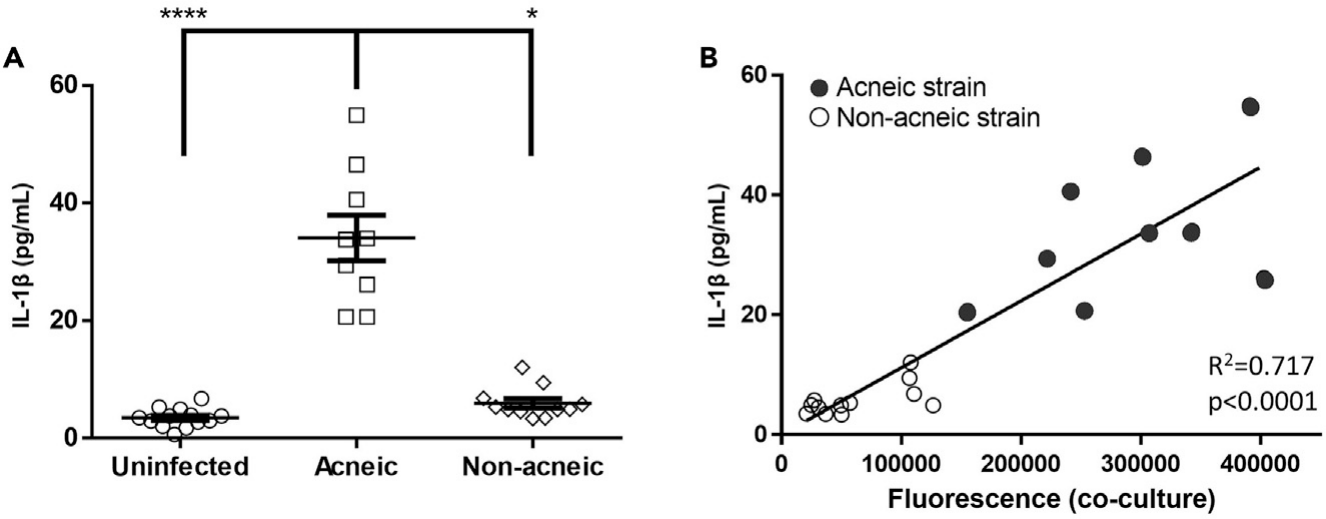
Correlation of *C. acnes* porphyrin production and induced IL-1β release (A) IL-1β released by HaCaT cells grown in the co-culture model infected with various *C. acnes* strains for 48 h. Data are mean ± SEM. (B) Correlation between *C. acnes*-produced porphyrins (fluorescence) and HaCaT IL-1β release. Error bars represent standard error of the mean. ∗p < 0.05, ∗∗∗∗p < 0.001

To elucidate if porphyrin production can be linked with IL-1β production, and with the association of certain *C. acnes* subgroups to acneic versus healthy skin, porphyrin production between both groups of *C. acnes* strains was quantified. First, the fluorescence (excitation: 405 nm, emission: 635 nm) in the supernatant of 24-h-old planktonic cultures of a selection of *C. acnes* strains (Table 1) was measured, as an indication for porphyrin production (Mancini and Imlay, 2015). Fluorescence was significantly higher in the supernatant of acne-associated *C. acnes* cultures compared with the supernatant of other strains (p < 0.0001) (Figure 6). Next, the fluorescence of the supernatant of the inserts in the HaCaT-sebocyte-*C. acnes* co-culture model, after 48 h of infection with the same *C. acnes* strains was measured, and we observed that the fluorescence was significantly higher after infection with acne-associated strains (p = 0.004) (Figure 6). To verify that this higher fluorescence was due to higher porphyrin production, fluorescence was measured in the supernatant of co-cultures after 48 h of infection with acne-associated strain HL053PA1 with or without 5-ALA or SA. Fluorescence was significantly higher in supernatant when 5-ALA was added (p < 0.0001) and significantly lower when SA was added (p = 0.0012) (Figure 6). To confirm these fluorescence measurements, porphyrins were extracted from a selection of acne-associated strains (HL043PA1, HL053PA1, and HL072PA1) as well as a selection of strains not associated with acne (HL002PA1, HL059PA1, and HL110PA3). The total amount of extracted porphyrins was quantified colorimetrically using a coproporphyrin III tetramethyl ester standard curve, and porphyrin production was found to be significantly higher in acne-associated *C. acnes* strains (p = 0.0014) (Figure 6). It had previously been observed that vitamin B12 supplementation increased porphyrin production in acne-associated strains but not in health-associated strains (Johnson et al., 2016), and this vitamin B12 supplementation resulted in the development of acne in a subset of the population only, confirming the importance of porphyrins in acne pathogenesis (Martínez De Espronceda Ezquerro et al., 2018; Balta and Ozuguz, 2014; Kang et al., 2015). Differences in porphyrin composition between strains were not investigated, as previous studies have reported similar compositions for different isolates (Johnson et al., 2016; Schaller et al., 2005). Finally, IL-1β production was measured in the supernatant of HaCaT cells grown in the co-culture model, and a significantly higher amount of IL-1β was produced by HaCaT cells infected with acne-associated *C. acnes* strains (p < 0.0001) (Figure 7). A significant correlation was found between porphyrin production by *C. acnes* and IL-1β release by the keratinocytes infected by these strains (R^2^ = 0.717, p < 0.0001) (Figure 7).

**Figure 8:**
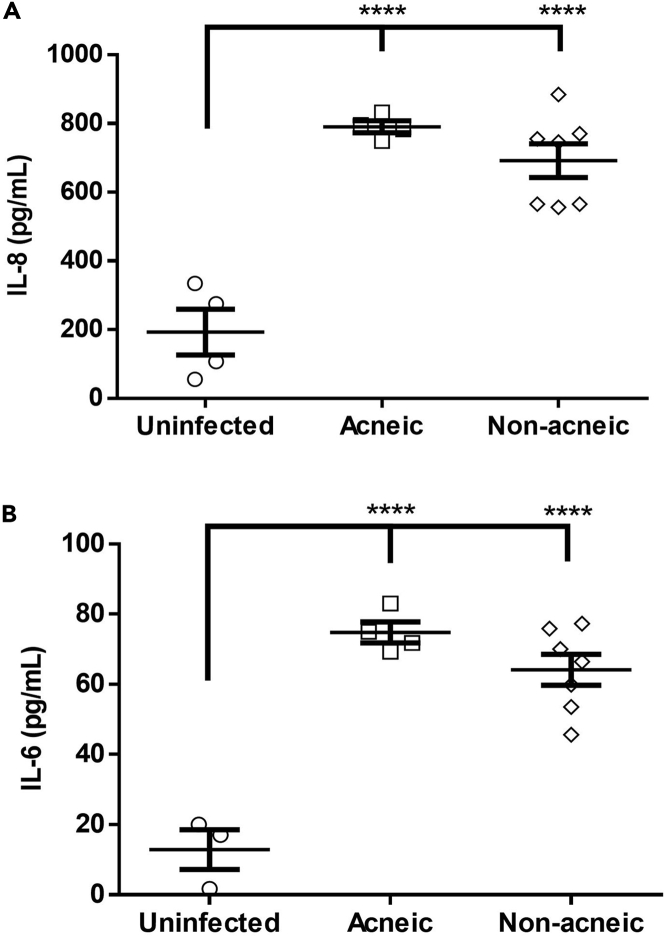
Inflammatory potential of *C. acnes* strains (A) HaCaT IL-8 release after 48 h of infection with *C. acnes*. (B) *C. acnes*-induced IL-6 release after 48 h of infection. Error bars represent standard error of the mean. ∗∗∗∗p < 0.001

Finally, all *C. acnes* strains tested were able to induce IL-6 and IL-8 production in HaCaT cells grown in the co-culture model, but there was no difference between both groups (Figure 8). Activation of Toll-like receptors (TLR) activates the nuclear factor-κB pathway resulting in the production of multiple pro-inflammatory cytokines including IL-6 and IL-8. This activation can be achieved through binding of LPS to TLR4 as well as binding of peptidoglycan to TLR2, two TLRs expressed by keratinocytes (Pivarcsi et al., 2003; Ondet et al., 2017; Niebuhr et al., 2010). As *C. acnes* is a Gram-positive bacterium, activation of TLR2 by peptidoglycan is likely to occur (Grange et al., 2009; Yuki et al., 2011). As peptidoglycan is found in all *C. acnes* strains, it is no surprise that this TLR2 activation (and subsequent IL-6 and IL-8 production) is observed for all strains.

### Conclusions

Combined our data show that acneic *C. acnes* strains produce high levels of porphyrins, which in turn leads to activation of the inflammasome via the induction of K^+^ efflux. Inflammasome activation requires two signals. The first signal leads to activation of the NF-κB pathway, resulting in the expression of pro-IL-1β and NLRP3 as well as the release of other pro-inflammatory cytokines. The second signal initiates the formation of the NLRP3 inflammasome and is often triggered by a disruption of cellular homeostasis (Lamkanfi and Dixit, 2014; Kelley et al., 2019). We propose that both acne-associated and non-acneic *C. acnes* strains prime the inflammasome through peptidoglycan, which is present in all *C. acnes* strains. However, acne-associated *C. acnes* strains produce more porphyrins than non-acneic strains, and these porphyrins can interact with the keratinocyte cell membrane resulting in the leakage of K^+^, thereby activating the NLRP3 inflammasome (Muñoz-Planillo et al., 2013). Our findings help explain why *C. acnes* is a commensal of the skin in some people and causes acne in others, and can aid in the development of new acne therapies. For example, components that inhibit porphyrin production in *C. acnes* (e.g., succinyl acetone) or sequester porphyrins (e.g., albumin) could potentially be beneficial in the treatment of acne. Using this strategy, only acne-associated strains are targeted without affecting the non-acneic strains or commensal organisms.

### Limitations of the study

While our study includes several important players in the pathogenesis of acne (keratinocytes, sebocytes, and *C. acnes*) it is still an *in vitro* study and confirmation in more relevant *in vivo*-like conditions would be a valuable addition. However, there are few acne animal models that accurately recapitulate the situation in humans (e.g., in many models infection is not needed for the development of acne). A recently developed model in which acne lesions are induced by the combined application of *C. acnes* and human sebum could be a valuable alternative, although this model has several drawbacks as well, including the requirement for intradermal administration of *C. acnes* and the relatively acute nature of the infection (Kolar et al., 2019). The use of skin biopsies is a possible alternative (Jahns and Alexeyev, 2014, 2016; Dagnelie et al., 2019; Jasson et al., 2013), but such biopsies are not easy to obtain and are small (i.e., would not necessarily always allow simultaneous analysis of microbial load and measurement of porphyrin and cytokine levels). Although the use of primary keratinocytes could potentially allow the incorporation of patient-specific factors, this is technically challenging.

Second, in our current setup no other microorganisms are included, although it seems likely that the skin microbiome will influence *C. acnes* and *C. acnes*-mediated inflammatory processes (Byrd et al., 2018; Dreno et al., 2020; Dagnelie et al., 2019; Jasson et al., 2013). For example, Wollenberg et al. (2014) showed that CPIII produced by *C. acnes* induces plasma-independent biofilm formation by *S. aureus*. In addition, skin bacteria will produce antimicrobial compounds to reduce the levels of competing microorganisms, e.g., various *Staphylococcus epidermidis* strains produce toxins (including bacteriocins) that inhibit *C. acnes* (Christensen et al., 2016), whereas several *C. acnes* strains produce the thiopeptide antibiotic cutimycin that has activity against staphylococci (Claesen et al., 2020). On top of that it has been shown that a reduced diversity in the skin-associated *C. acnes* population (so-called 'phylotype diversity loss') leads to an increased response of the innate immune system against *C. acnes* (Dagnelie et al., 2019). These data clearly show that in order to obtain a complete picture of factors involved in the pro- or anti-inflammatory activity of *C. acnes*, the presence and activity of other microorganisms needs to be considered as well. Although this was outside the scope of the present study, we believe our model would be well-suited to investigate this in the future.

Third, little is known about concentration of porphyrins inside the sebaceous gland and in sebum, and it remains to be seen whether the concentrations of porphyrins used in the present study are physiologically relevant (Borelli et al., 2006; Gannesen et al., 2019).

Finally, *C. acnes* produces other potential virulence factors (including proteases, lipases, and CAMP factor), and the production of these can vary between strains (Coenye et al., 2007; Spittaels and Coenye, 2018). Despite the observed strong link between porphyrin production and inflammation, it cannot be ruled out that the differential production of other virulence factors contributes to the differences in inflammatory response observed in the present study. However, the observation that similar results are obtained with cells and with the porphyrin extract from these cells suggests that the influence of other virulence factors would be small.

## STAR★methods

### Key resources table

**Table:** 

REAGENT or RESOURCE	SOURCE	IDENTIFIER
**Bacterial and virus strains**		

*Cutibacterium acnes*	BEI Resources	HL072PA1
*Cutibacterium acnes*	BEI Resources	HL036PA1
*Cutibacterium acnes*	BEI Resources	HL005PA1
*Cutibacterium acnes*	BEI Resources	HL038PA1
*Cutibacterium acnes*	BEI Resources	HL045PA1
*Cutibacterium acnes*	BEI Resources	HL053PA1
*Cutibacterium acnes*	BEI Resources	HL056PA1
*Cutibacterium acnes*	BEI Resources	HL043PA2
*Cutibacterium acnes*	BEI Resources	HL086PA1
*Cutibacterium acnes*	BEI Resources	HL002PA1
*Cutibacterium acnes*	BEI Resources	HL027PA1
*Cutibacterium acnes*	BEI Resources	HL030PA2
*Cutibacterium acnes*	BEI Resources	HL059PA1
*Cutibacterium acnes*	BEI Resources	HL059PA2
*Cutibacterium acnes*	BEI Resources	HL050PA2
*Cutibacterium acnes*	BEI Resources	HL060PA1
*Cutibacterium acnes*	BEI Resources	HL082PA2
*Cutibacterium acnes*	BEI Resources	HL110PA3
*Cutibacterium acnes*	BEI Resources	HL110PA4
*Cutibacterium acnes*	BEI Resources	HL110PA4

**Chemicals, peptides, and recombinant proteins**		

5-aminolevulinic acid	Sigma	Cat#A3785
Succinyl acetone	Sigma	Cat#D1415
Coproporphyrin I	Sigma	Cat#258784
PBFI AM	Abcam	Cat#ab142804

**Critical commercial assays**		

Human IL-6 ELISA Kit	BioLegend	Cat#430507
Human IL-8 ELISA Kit	BioLegend	Cat#431507
Human IL-1β ELISA Kit	BioLegend	Cat#437007
FAM FLICA caspase-1 assay	ImmunoChemistry Technologies	Cat#97
Lactate dehydrogenase activity assay kit	Sigma	Cat#MAK066-1KT

**Experimental models: Cell lines**		

HaCaT keratinocyte cell line	Boukamp et al. (1988)	CLS Cell Lines Service GmbH
SZ95 sebocytes	Zouboulis et al., 1999	C.C. Zouboulis

**Software and algorithms**		

ImageJ	NIH	https://imagej.nih.gov/ij/

**Other**		

Anaerogen Compact system	Oxoid	Cat#AN0010
Gaspak EZ system	BD	Cat#DIFC260683

### Resource availability

#### Lead contact

Further information and requests for resources and reagents should be directed to and will be fulfilled by the Lead Contact, Tom Coenye (tom.coenye@ugent.be).

#### Materials availability

This study did not generate new unique reagents.

#### Data and code availability

This study did not generate/analyze datasets or code.

### Experimental model and subject details

#### Bacterial cultures

The *C. acnes* strains used in this study are listed in Table 1 and were obtained from BEI Resources, NIAID, NIH as part of the Human Microbiome Project (Manassas, VA, USA). Strains were grown on Reinforced Clostridial Agar (RCA; LabM, Heywood, UK) at 37°C under anaerobic conditions generated with the Anaerogen Compact system (Oxoid, Aalst-Erembodegem, Belgium) or the Gaspak EZ system (BD, VWR, Leuven, Belgium) for at least three days.

#### Cell culture and co-culture model

The HaCaT spontaneously immortalized human keratinocyte cell line (Boukamp et al., 1988) was cultivated in Dulbecco's Modified Eagle Medium (DMEM; Gibco, Life Technologies Corporation, NY, USA) supplemented with 10% fetal bovine serum (FBS; Gibco) and 1% penicillin/streptomycin (pen/strep; 100 UI/mL, Sigma-Aldrich, Steinheim, Germany) at 37°C in a humidified incubator with 5% CO_2_. HaCaT cells were seeded in a 24-well cell culture plate (Greiner Bio-One, Frickenhausen, Germany) at a density of 2.5 x 10^4^ cells per well and incubated until confluency while fresh medium containing pen/strep was added every two days. Before the experiments started, the cells were washed with phosphate buffered saline (PBS, Gibco) and medium without either pen/strep or FBS was added. The SZ95 immortalized human sebaceous gland cell line (Zouboulis et al., 1999) was cultivated in Sebomed basal medium (Biochrom, Berlin, Germany) supplemented with 10% FBS, 1% pen/strep, 1 mM CaCl_2_, and 5 ng/mL recombinant human epidermal growth factor (rhEGF; Thermo-Fisher, MA, USA). A co-culture model was established using SZ95 sebocytes and HaCaT cells. To this end, SZ95 cells were seeded in a 24-well cell culture plate (Greiner Bio-One) at a density of 2.5 x 10^4^ cells per well in combination with supplemented Sebomed medium, which was refreshed every two days. HaCaT cells were seeded in 24-well plate inserts (ThinCert, pore diameter 0.4 μm, Greiner Bio-One) and cultivated in supplemented DMEM as described above. After 5 days, both cell lines were washed with PBS and the inserts, containing the HaCaT cells, were transferred to the wells containing the SZ95 cells. Subsequently, supplemented Sebomed medium was added and the plates were incubated for an additional two days of co-culture. After a total of 7 days, the medium was removed from the wells and inserts, the cells washed with PBS, and Sebomed basal medium supplemented with 1 mM CaCl_2_ and 5 ng/mL rhEGF was added. We have previously shown that in these conditions there are no differences in cell viability 24 and 48h after infection with different *C. acnes* strains and that HaCaT cell dead overall was very low (4-6%) and not significantly different from the uninfected control (Spittaels et al., 2020).

### Method details

#### Infection of the keratinocytes

Planktonic cultures of *C. acnes*, grown anaerobically for 24h in Sebomed basal medium, were centrifuged at 3500 rpm for 5 min (Eppendorf centrifuge 5804 R, Eppendorf, Hamburg, Germany) after which the bacterial pellet was washed and resuspended in PBS. These bacterial suspensions were used to infect the HaCaT cells, grown as a monolayer and in the insert of the co-culture model, at a multiplicity of infection (MOI) of 10:1 (Spittaels et al., 2020). The infected cell culture plates were incubated anaerobically using the Anaerogen compact system at 37°C for 48 h. A schematic overview of the model is shown in Figure S3.

#### Porphyrin production

Porphyrins were extracted from bacterial pellets as described previously (Mancini and Imlay, 2015). In short, planktonic cultures of *C. acnes* were grown in Reinforced Clostridial Medium (RCM; LabM) and incubated anaerobically for 4 days. The cultures were then centrifuged for 10 min at 5,000 rpm and 4°C (Eppendorf centrifuge 5804 R, Eppendorf, Hamburg, Germany) and the bacterial pellets were washed in 10 mL pre-chilled 0.05M TRIS - 2mM EDTA (pH 8.2; Sigma Aldrich). To compare porphyrin production between different strains, the washed bacterial pellets were resuspended in the TRIS-EDTA buffer and adjusted to an optical density of 1.0 at 590 nm. Next, the suspensions were centrifuged again and the bacterial pellets were resuspended in 5 mL ethyl acetate/acetic acid (4:1, V/V). Cells were subsequently lysed by sonication on ice for 30 min, after which cellular debris was removed through centrifugation for 5 min at 5,000 rpm and 4°C. The porphyrin-containing supernatant was transferred to a new tube and washed thrice in 1 mL distilled H_2_O; porphyrins were finally solubilized in 1.5 M hydrochloric acid (Sigma). The concentration of total porphyrins extracted was determined by measuring the absorbance of the extracts at 405 nm using an EnVision Multilabel Plate Reader (Perkin Elmer, Waltham, MA, USA) and comparing this with absorbances obtained with a coproporphyrin III tetramethyl ester (Sigma) standard curve.

#### Differences in porphyrin production between strains

In order to rapidly quantify relative differences in porphyrin production between strains in planktonic cultures (in Sebomed basal medium) and after 48 h infection in the co-culture model, fluorescence was measured in the supernatant. To this end, the supernatant was collected in Eppendorf tubes, centrifuged for 20 min at 5,000 rpm (Eppendorf centrifuge 5427 R, Eppendorf) and filtered through a 0.2 μm pore filter (GE Healthcare Life Sciences, MA, USA). Next, 100 μL of the supernatant was transferred to a black MTP (Thermo-Fisher) and fluorescence was measured using an EnVision Multilabel Plate Reader (Perkin Elmer) (excitation: 405 nm, emission: 635 nm). To induce or inhibit the production of porphyrins, a substrate or inhibitor of the porphyrin pathway respectively were added during infection. The concentration of the substrate (5-aminolevulinic acid; 5-ALA) was 0.2 mg/mL and of the inhibitor (succinyl acetone; SA) 0.5 mM. From previous experiments we know that there are no meaningful differences in growth between the different *C. acnes* strains (Spittaels et al., 2020) which were in early stationary phase after 48h (Figure S4).

#### Identification and relative quantification of porphyrins

Identification and relative quantification of different types of porphyrins in the extract was accomplished by liquid chromatography followed by high-resolution mass spectrometry (tandem quadrupole time-of-flight) based on a previously described untargeted screening method (Thoren et al., 2016). To separate the different porphyrins in the extract, an Agilent 1290 Infinity LC system and Phenomenex Kinetex C18-column (2.6 μm, 3 x 50 mm) maintained at 30°C, were utilized. A binary mobile phase at a flow rate of 400 μL/min was used comprising of (A) 0.05% formic acid in 5 mmol/L ammonium formate in water and (B) 0.05% formic acid in methanol/acetonitrile (50:50, V/V). Reagents were at least analytical grade and purchased from Sigma Aldrich or Biosolve (Valkenswaard, The Netherlands). The mobile phase started at 40% B which increased linearly to 80% over 10 min. Next, the column was washed at 100% B for 2 min and equilibrated again at 2% B for 2 min. Coproporphyrin I (Sigma) was used as a standard and it was diluted to a final concentration of 1 ng/mL with A/B (50:50, V/V). The extract was diluted in a similar way. Injection volumes were 10 and 20 μL, respectively. Identification of the specific porphyrins was accomplished based on the predicted m/z values utilizing a 5600 QTOF system (AB Sciex, MA, USA) coupled to an electrospray ionization (ESI) source in the positive mode. Information-dependent acquisition was used scanning in both the TOF-MS and product ion mode from 300-750 Da. The mass spectrometry parameters were as follows: ion source gas 1: 30 psi, ion source gas 2: 30 psi, curtain gas: 25 psi, temperature: 500°C, ion spray voltage: 5500 V, declustering potential 100 V, and collision energy 35 V. The Analyst TF 1.7.1 software was used for data acquisition, Peakview 2.2 and MasterView 1.1 were used for data analysis.

#### Cytokine production

In order to investigate the activation of the inflammasome by the porphyrins, HaCaT cells were first primed with 0.1 mg/mL LPS (Sigma) for 4h. While LPS is not produced by *C. acnes*, we preferred using LPS over heat-killed *C. acnes*, or peptidoglycan or lipoproteins from *C. acnes*, as such preparations potentially could also contain an (unknown) amount of porphyrins. After priming, the cells were washed with PBS, and Sebomed basal medium supplemented with 1 mM CaCl_2_ and 5 ng/mL rhEGF was added. The porphyrin-containing extract was neutralized to a pH of approximately 7.0 using 1.5 M NaOH (Sigma) right before stimulation of the HaCaT cells. After 48 h infection or stimulation with the neutralized porphyrin extract, the supernatant of the HaCaT cells was collected in Eppendorf tubes and filtered as described previously. IL-6, IL-8, and IL-1β cytokine concentrations were determined using sandwich ELISA assays (BioLegend, CA, USA) according to the manufacturer’s instructions.

#### Caspase-1 activity

Caspase-1 activation was measured using a FAM FLICA caspase-1 assay (FAM-YVAD-FMK, ImmunoChemistry Technologies, MN, USA). HaCaT monolayer cells were cultivated for 5 days in 24-well cell culture plates in supplemented DMEM containing pen/strep. The cells were washed with PBS and the medium was changed to supplemented Sebomed basal medium for an additional two days. Before infection or stimulation, the cell culture medium was removed, cells were washed with PBS, and Sebomed medium only containing 1 mM CaCl_2_ a 5 ng/mL rhEGF was added. Cells were infected with *C. acnes* at a MOI of 10:1 or stimulated with neutralized extract (containing multiple porphyrins with a total porphyrin concentration of 2.5 μM or 1 μM) for 48 h as described above. Subsequently, the supernatant was removed, the cells washed with PBS, and 290 μL Sebomed basal medium (supplemented only with CaCl_2_ and rhEGF) and 10 μL of the 30X FAM FLICA caspase-1 reagent were added. The plates were then incubated for 1 h, after which the cells were rinsed thrice with the wash buffer included in the kit, and then imaged using the EVOS FL Auto Imaging System (Life technologies, Ca, USA), equipped with a 10x objective (FAM FLICA: excitation: 470 nm, emission: 510 nm, final magnification: 300x). Fluorescence was quantified using the ImageJ software (National Institutes of Health, MD, USA). The FAM FLICA stained HaCaT keratinocytes were subsequently harvested for flow cytometry. In brief, cells were treated with 0.05% EDTA (Sigma) in PBS for 10 min at 37°C, followed by 1 min trypsinization (0.025% trypsin, Sigma). The population of active caspase-1 containing HaCaT cells was determined by measuring fluorescence using the Attune NxT flow cytometer (Thermo Fisher).

#### Propidium iodide staining

HaCaT cells were seeded at a density of 2.5 x 10^4^ cells per well in a black 24-well MTP with glass bottom (Greiner Bio-One) and cultured as described earlier. After 7 days, the HaCaT cells were stimulated with the porphyrin extract. Changes in cellular permeability were assessed using the fluorescent dye propidium iodide (PI; Life Technologies). 4 μL of a 20 mM PI solution in DMSO was mixed with 996 μL PBS. After a 30 min treatment with the porphyrin extract, the cells were washed with PBS following the removal of the supernatant. 500 μL of the PI solution was added to the wells and the plate was incubated for 10 min at 37°C, protected from light. Afterwards, the dye was removed and the cells were washed with 500 μL PBS. The EVOS FL Auto Imaging System equipped with a 10x objective (excitation: 531 nm, emission: 593 nm, final magnification: 300x) was used for fluorescence microscopy. Fluorescence was quantified using the ImageJ software (National Institutes of Health, MD, USA).

#### PBFI-AM staining

Potassium efflux was confirmed by investigating the intracellular K^+^ content of the cells using the cell permeable, fluorophore PBFI-AM (Abcam, Cambridge, United Kingdom). After 48 h infection or treatment with the porphyrin extract, supernatant was removed and the cells were washed with Hanks balanced salt solution (HBSS; Gibco). 400 μL loading buffer (PowerLoad, Invitrogen) was added to the wells and 10 μM PBFI-AM and 10 μM Pluronic F-127 (Invitrogen) were included per well, after which the plate was incubated, protected from light, for 2h at 37°C. Afterwards, the loading buffer was removed and the cells were washed with HBSS. Imaging was accomplished using the EVOS FL Auto Imaging System equipped with a 10x objective (excitation: 357 nm, emission: 447 nm, final magnification: 300x). Fluorescence intensity was measured using the ImageJ software.

#### Host cell viability

Viability of the HaCaT cells was measured in the co-culture model using a lactate dehydrogenase activity assay kit (LDH assay; Sigma Aldrich) according to the manufacturer’s instructions.

### Quantification and statistical analysis

Values represent mean ± standard error of the means (SEM) obtained from a minimum of three biological replicates. Data were analyzed using SPSS Statistics version 25. Independent-Samples T-tests or Mann-Whitney U tests were used, depending on the normality of the data set, to compare two groups. A one-way analysis of variance (ANOVA) or a Kruskal-Wallis test was performed to compare more than two groups. Mean differences were considered statistically significant at a p-value ≤ 0.05.
