# Supplementary material for: Porphyrins produced by acneic Cutibacterium acnes strains activate the inflammasome by inducing K+ leakage
Source: iScience. 2021 May 21;24(6):102575. doi: 10.1016/j.isci.2021.102575 (PMC8188554; doi:10.1016/j.isci.2021.102575)

iScience, Volume *24*

Supplemental information

Porphyrins produced by acneic *Cutibacterium acnes* strains activate the inflammasome by inducing K^+ leakage

Karl-Jan Spittaels, Katleen van Uytfanghe, Christos C. Zouboulis, Christophe Stove, Aurélie Crabbé, and Tom Coenye

Figure S1. Related to Figure 4. K^+ efflux as determined by quantification of PBFI-AM fluorescence after 30 min stimulation of HaCaT monolayer cells with 1 µM CPI, CPIII, PPIX or the extracted porphyrins. Error bars represent standard error of the mean. ** $p<0.01$, *** $p<0.05$.

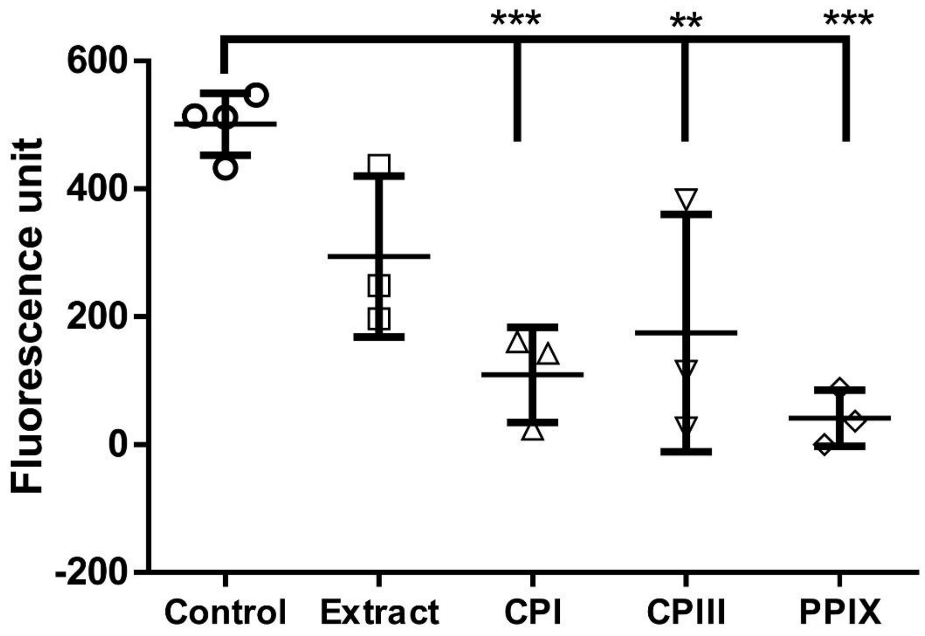

Figure S2. Related to Figure 4. Cell viability after 30 min exposure to extracted porphyrins at 2.5 µM, 1.0 µM, and 0.5 µM, measured by determination of LDH release and expressed as the % of dead cells compared to 100% lysed cells using 1 %Triton X-100. Data presented are mean ± SEM.

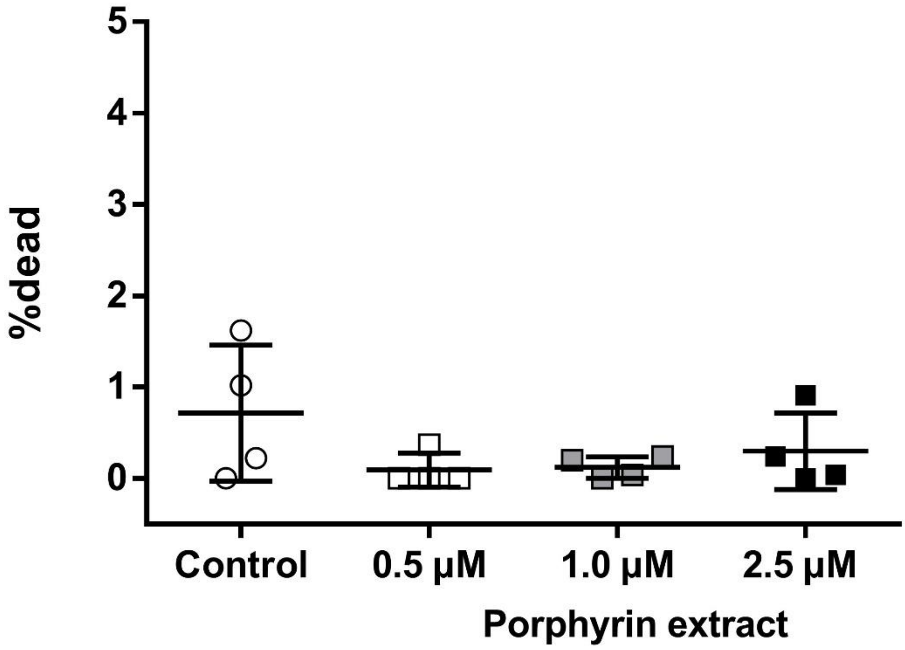

Figure S3. Schematic overview of the keratinocyte-sebocyte co-culture model. Related to STAR Methods. Sebocytes are cultured on bottom of a 24 well MTP while keratinocytes are cultured in well inserts separately for 5 days. Both cultures are combined for 2 days after which *C. acnes* is added in the inserts.

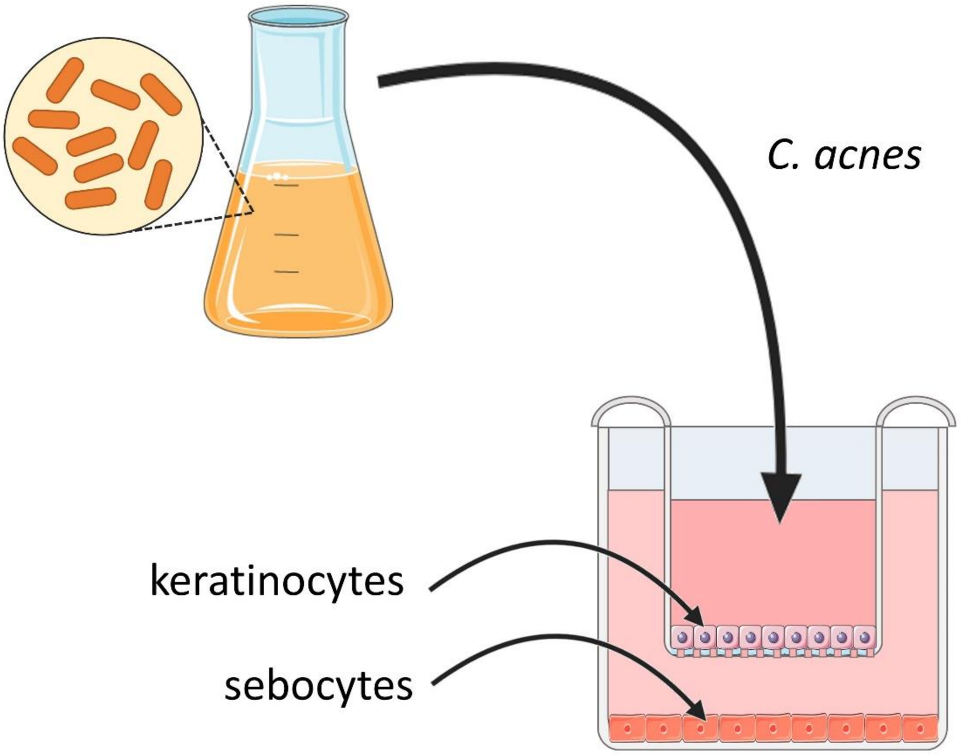

Figure S4. Related to STAR Methods. Optical densities (OD, 590 nm) of 48h old planktonic *C. acnes* cultures grown in Reinforced Clostridial Medium. Top: data for individual strains. Bottom: average per phylotype (error bar: standard deviation).

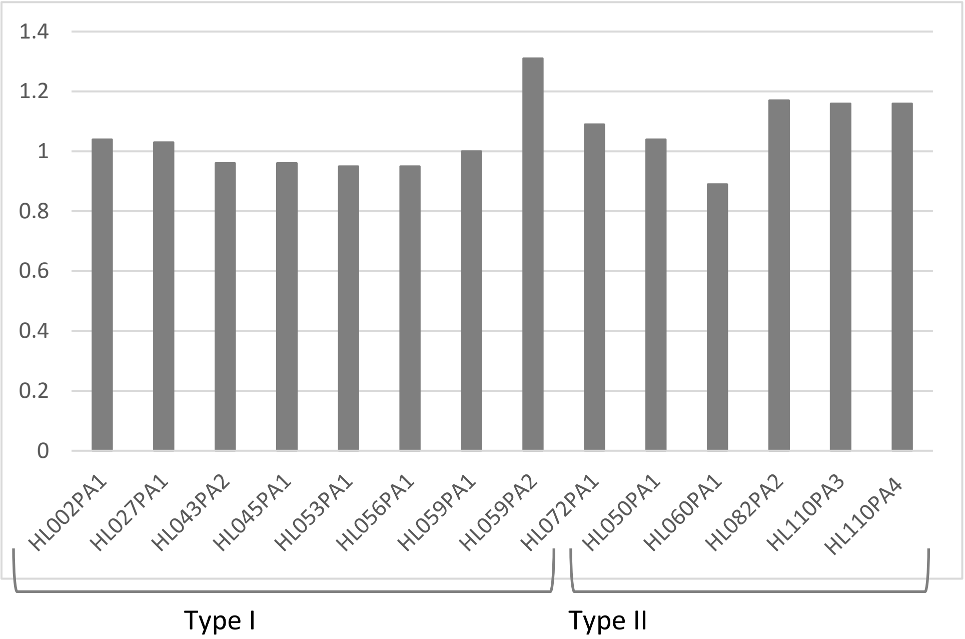

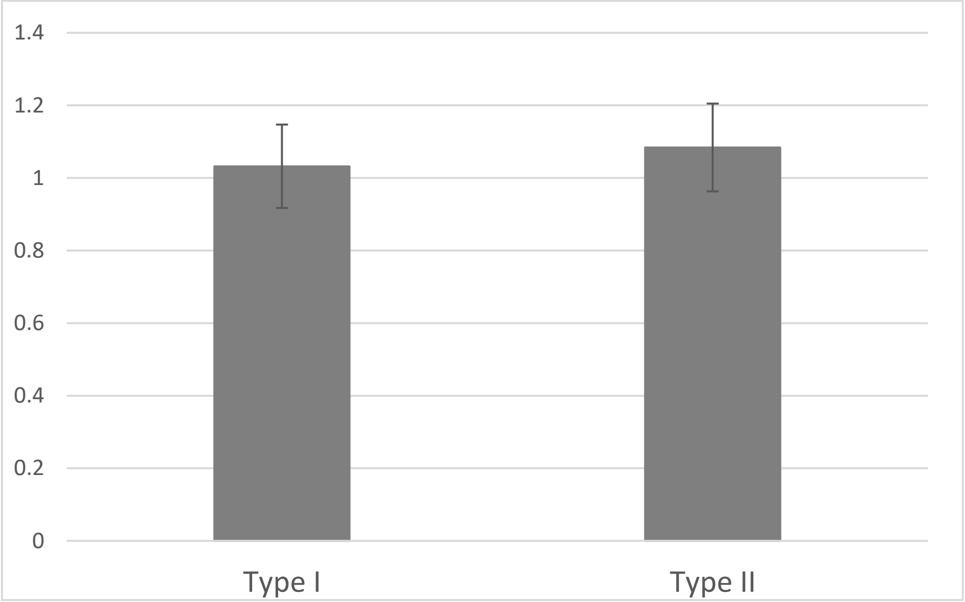

Supplement: Document S1. Figures S1–S4 [file mmc1.pdf]
